# Origins and Long-Term Patterns of Copy-Number Variation in Rhesus Macaques

**DOI:** 10.1093/molbev/msaa303

**Published:** 2020-11-23

**Authors:** Gregg W C Thomas, Richard J Wang, Jelena Nguyen, R Alan Harris, Muthuswamy Raveendran, Jeffrey Rogers, Matthew W Hahn

**Affiliations:** 1 Division of Biological Sciences, University of Montana, Missoula, MT, USA; 2 Department of Biology, Indiana University, Bloomington, IN, USA; 3 Department of Computer Science, Indiana University, Bloomington, IN, USA; 4 Human Genome Sequencing Center, Baylor College of Medicine, Houston, TX, USA; 5 Department of Molecular and Human Genetics, Baylor College of Medicine, Houston, TX, USA

**Keywords:** copy-number variation, de novo mutation, genomics, rhesus macaque, pedigree sequencing, structural variation

## Abstract

Mutations play a key role in the development of disease in an individual and the evolution of traits within species. Recent work in humans and other primates has clarified the origins and patterns of single-nucleotide variants, showing that most arise in the father’s germline during spermatogenesis. It remains unknown whether larger mutations, such as deletions and duplications of hundreds or thousands of nucleotides, follow similar patterns. Such mutations lead to copy-number variation (CNV) within and between species, and can have profound effects by deleting or duplicating genes. Here, we analyze patterns of CNV mutations in 32 rhesus macaque individuals from 14 parent–offspring trios. We find the rate of CNV mutations per generation is low (less than one per genome) and we observe no correlation between parental age and the number of CNVs that are passed on to offspring. We also examine segregating CNVs within the rhesus macaque sample and compare them to a similar data set from humans, finding that both species have far more segregating deletions than duplications. We contrast this with long-term patterns of gene copy-number evolution between 17 mammals, where the proportion of deletions that become fixed along the macaque lineage is much smaller than the proportion of segregating deletions. These results suggest purifying selection acting on deletions, such that the majority of them are removed from the population over time. Rhesus macaques are an important biomedical model organism, so these results will aid in our understanding of this species and the disease models it supports.

## Introduction

Mutations are an important source of genetic variation, and can have both immediate effects on individual phenotypes and lasting impacts on genome evolution. Understanding how mutations arise and spread through a population in the short-term can therefore aid our understanding of disease, while understanding their effects in the long-term aid our understanding of evolution in populations and species. Recent work in humans and other primates have unveiled patterns of mutation for single-nucleotide variants (SNVs) using pedigrees of related individuals. For instance, studies in primates have found a strong paternal age effect on the number of de novo single-nucleotide mutations: older fathers tend to pass on more mutations (Kong et al. 2012; Venn et al. 2014; Jonsson et al. 2017; Thomas et al. 2018). This is likely due to a combination of errors accruing from both ongoing spermatogenesis and unrepaired DNA damage. However, no such paternal age effect has been found among de novo deletions and duplications (also known as copy-number variants, or CNVs) in humans (MacArthur et al. 2014; Kloosterman et al. 2015; Brandler et al. 2016; Girard et al. 2016), though the origin of CNVs have been studied less often than single-nucleotide mutations (Sebat et al. 2007; Itsara et al. 2010; Schrider et al. 2013; MacArthur et al. 2014; Kloosterman et al. 2015; Brandler et al. 2016; Girard et al. 2016; Werling et al. 2018).

The frequency of CNVs among lineages and the density of CNVs along the genome have been found to be highly variable among primates (Fortna et al. 2004; Jiang et al. 2007; Sudmant et al. 2010; Gazave et al. 2011), with CNV hotspots in multiple species having been described (Perry et al. 2006, 2008; Gokcumen et al. 2011). Duplications in genic regions have been found to outnumber deletions in many lineages when comparing closely related species (Fortna et al. 2004; Dumas et al. 2007; Sudmant et al. 2013), possibly indicating differential effects of natural selection on gene duplications versus deletions. However, recent whole-genome studies in humans point to a different pattern in nongenic regions, with deletions far outnumbering duplications (Brandler et al. 2016). In order to determine whether such patterns are specific to humans, or are representative of the joint effects of mutational input and selection on the long-term survival of duplications and deletions, we require fine-scale studies in additional species.

Rhesus macaques (*Macaca mulatta*) are a widely used model organism, especially for studies of human diseases. Understanding the underpinnings of genetic variation in this species may help to enhance studies on existing or new disease models, in addition to aiding our understanding of the genetic basis of evolutionary change. Previous studies of rhesus macaque CNVs have used array-based comparative genomic hybridization (aCGH) to detect events and have found that the frequency of duplications either matches or exceeds that of deletions (Lee et al. 2008; Gokcumen et al. 2011), whereas more recent studies show a bias toward deletions (Gokcumen et al. 2013). However, aCGH methods are limited in their detection of short deletions and duplications (Medvedev et al. 2009; Zarrei et al. 2015), and may have included the insertion of transposable elements (which we do not consider here). Patterns of variation in CNVs shorter than the detectable limit by aCGH remain uncharacterized. Read-based methods—which use read depth, read orientation, discordance of paired-end reads from a reference genome, or a combination of these signals (reviewed in Medvedev et al. 2009; Zhang et al. 2019)—will help to clarify patterns of duplication and loss.

Here, we use deep sequencing of 32 rhesus macaques including 14 sire-dam-offspring trios to uncover patterns of copy-number mutation and variation in this species. We find that, contrary to aCGH studies, deletions make up the vast majority of polymorphic CNVs within rhesus macaques. Using unrelated individuals, we find that patterns of segregating CNVs are similar between macaques and humans. By sequencing parent–offspring trios, we are also able to investigate the occurrence of de novo CNVs. We find that the number of de novo CNVs per generation is less than one per genome in both macaques and humans, and that parental age has no detectable effect on the rate of these types of mutations in either species. Finally, we compare patterns of deletions and duplications in our sample to those of long-term gene gains and losses along the lineage leading to macaques from their last common ancestor with baboons (genus *Papio*). Interestingly, whereas deletions make up the vast majority of polymorphisms in our sample, the number of genes gained and lost along the macaque lineage is roughly equal. These patterns give us a first look at the origins of copy-number variation using whole-genome sequencing in a nonhuman primate, and will help improve modeling of these types of mutations in relation to both disease prediction and evolutionary analyses.

## Results

### Patterns of Copy-Number Variation in Rhesus Macaques

**Fig. 1.**
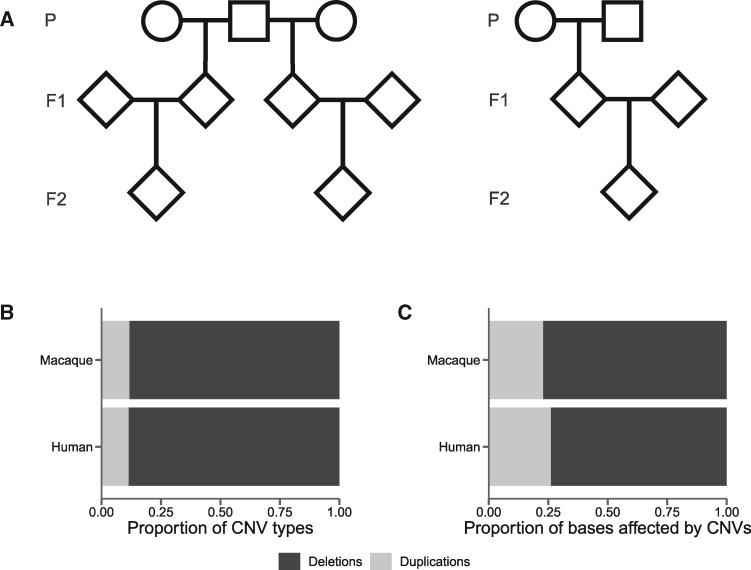
(*A*) Pedigrees of sequenced macaques. The 14 trios were contained within three families similar to the one on the left, and one family similar to the one on the right. (*B*) The proportion of CNV types (deletions or duplications) and (C) bases affected by CNVs for rhesus macaques compared with humans.

We identified CNVs by sequencing the whole genomes of 32 Indian-origin rhesus macaques (fig. 1*A*;supplementary table S1, Supplementary Material online; Wang et al. 2020). We mapped the reads from these samples to the reference macaque genome (rheMac8, also called Mmul_8.0.1, downloaded April 12, 2018) and identified CNVs based on split and discordant read patterns using Lumpy (Layer et al. 2014), SVtyper (Chiang et al. 2015), and SVtools (Larson et al. 2018). We then filtered these calls by read-depth using Duphold (Pedersen and Quinlan 2019). These methods focus on identifying novel deletions and duplications of genomic regions, and repeat regions are explicitly removed from these analyses. As a consequence, we do not consider the patterns of mobile element insertions across the macaque genome. In total, we found 3,214 deletions and 432 duplications among these 32 individuals relative to the reference genome, meaning that roughly 88% of variants segregating in our sample are deletions (fig. 1*B* andsupplementary table S2, Supplementary Material online). Early studies of CNVs in rhesus macaques found roughly half of events to be deletions and half to be duplications (Gokcumen et al. 2011), whereas later comparative approaches observe a similar bias toward deletions (Gokcumen et al. 2013) and an excess of deletions is broadly observed throughout studies of structural variation in vertebrates (supplementary table S3, Supplementary Material online). Exceptions include studies based on read-depth, one of which is comparable to this study and shows an excess of duplicated bases in both rhesus macaques and humans (Brasó-Vives et al. 2020). The patterns inferred using read-depth alone are contradictory to the ones inferred here using read-pair orientation, highlighting the possibility of technical artifacts that could bias observed patterns in CNV studies and a critical need for comparisons among read-based CNV calling methods. Regarding early aCGH studies, one possibility for the difference between them and more recent read-based studies is that the former could not resolve events shorter than a few kilobases (minimum length 3,518 bases), whereas the read-based methods employed here can. This contrast from an increased level of resolution is consistent with studies in *Drosophila melanogaster* that found a bias toward deletions only for short events (Schrider et al. 2013). Other studies in humans show a similar bias toward detection of deletions, mostly driven by a lack of resolution for duplications less than 5000-bp long (Mills et al. 2011; Sudmant et al. 2015). These studies make sensitivity estimates on their CNV calls of 88% for deletions and 65% for duplications (Sudmant et al. 2015), indicating that the excess in deletion calls may actually be due to a higher rate of false negatives for duplications. However, even after correcting for these sensitivity estimates in our call-set, we still find an excess of deletions in both the macaque and human data. Specifically, these sensitivity estimates imply we are missing roughly 438 deletions and 233 duplications from our call-set, inclusion of which would still result in an excess of about 3,000 deletions.

We find that macaque CNVs are distributed across all chromosomes, but unevenly, with some stretches completely void of events and others where CNVs seem to be enriched (supplementary fig. S1, Supplementary Material online). Contrary to previous studies in rhesus macaques (Lee et al. 2008), we find that the number of CNVs on a chromosome is strongly correlated with the length of the chromosome (supplementary fig. S2, Supplementary Material online). This may again be the result of the increased resolution in our study. We also observe some clustering in the telomeric regions (supplementary fig. S1, Supplementary Material online). This telomeric clustering is consistent with the duplication maps of the macaque genome (Gibbs et al. 2007) and the human genome (Bailey et al. 2001; Fortna et al. 2004; Zarrei et al. 2015), and is likely driven by the higher concentration of transposable elements in these regions, which mediates higher levels of nonallelic homologous recombination (i.e., unequal crossing-over).

We used published CNVs from a sample of 235 humans (Brandler et al. 2016) to study the similarities and differences between primate species. Although these calls do include mobile element insertions, we restrict our comparisons to the deletion and duplication calls to coincide with our analyses. We find that the proportions of segregating deletions and duplications are not significantly different between the two species (fig. 1*B*; χ^2^ = 0.34, df = 1, *P *= 0.56). Given the observed bias toward deletions, it is unsurprising that both species have a higher proportion of bases deleted than duplicated (fig. 1*C*). The average individual in our macaque sample is heterozygous for 1,384 CNVs that delete 3,121,308 bp and duplicate 481,767 bp.

**Fig. 2.**
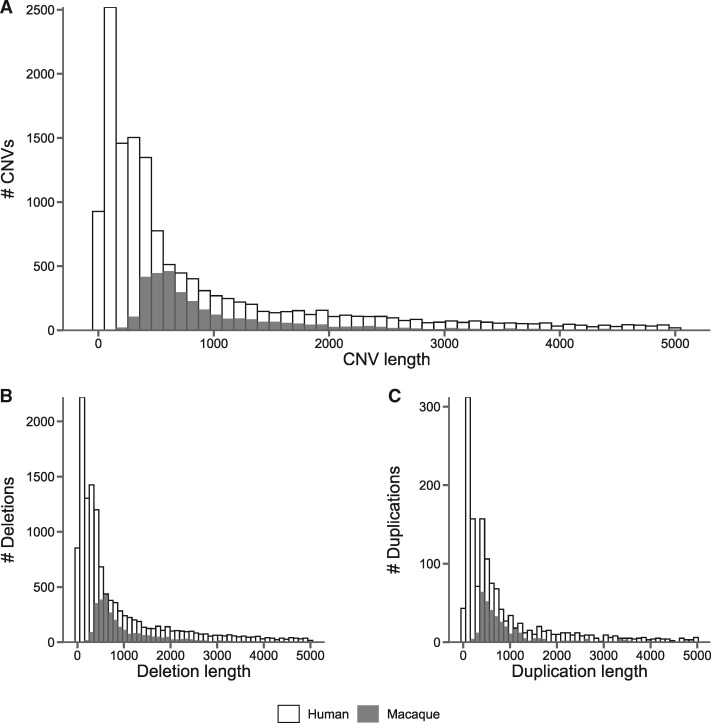
Length distributions of CNVs shorter than 5,000 bases and excluding all calls between 275 and 325 bp long as possible unannotated Alu elements. Distributions shown for (*A*) all CNVs, (*B*) deletions only, and (*C*) duplications only. Values are overlaid, with macaque bins in front of human bins. All bins start at 0 on the *y* axis.

CNVs in macaques have an average length of 3,615 bases, with duplications (mean length 6,990 bp; min length 138 bp; max length 97,301 bp) being longer than deletions (mean length 3,161 bp; min length 40 bp; max length 98,035 bp). Compared with humans, macaques have longer CNVs on an average (fig. 2*A*; Kolmogorov–Smirnov *D *= 0.43, *P *≪* *0.01) and this pattern holds for both deletions (fig. 2*B*; Kolmogorov–Smirnov *D *=* *0.43, *P *≪* *0.01) and duplications (fig. 2*C*; Kolmogorov–Smirnov *D *=* *0.37, *P *≪* *0.01). This pattern is mostly driven by the lack of shorter CNVs detected in macaques (supplementary fig. S3, Supplementary Material online). Though both analyses explicitly exclude long repeat regions, we also filter out possibly unannotated classes of shorter families of transposons, such as Alu elements, by excluding all CNV calls around 300 bp long—the average length of an Alu (Quentin 1992). Including these calls does not affect the comparisons between humans and macaques (supplementary fig. S6, Supplementary Material online). One possible explanation for the shift in average length of CNVs could be the observed lower recombination rate in rhesus macaques compared with humans (Xue et al. 2016), meaning there are fewer opportunities to break-up initial CNVs into smaller chunks by subsequent recombination events. However, it remains unclear whether this shift in CNV length distributions between macaques and humans is a true biological phenomenon, which would point to some change in the underlying CNV mechanism, or simply reflects our inability to detect very small variants in macaques. We took every effort to eliminate methodological bias between the macaque CNV calls and the human CNV data set. In their paper, Brandler et al. (2016) use several different CNV calling and genotyping methods. We have restricted our comparisons to CNVs called with the same methods we employed for the macaque data, namely CNVs called with Lumpy (Layer et al. 2014) and genotyped with SVtyper (Chiang et al. 2015). To test the effects of different CNV calling methods and filtering steps, we made the same comparison between macaque and human CNV lengths while using the full human data set (supplementary fig. S4, Supplementary Material online) and without filtering the macaque CNV calls (supplementary fig. S5, Supplementary Material online). Regardless of the partitioning method used, we still observe that macaques have, on an average, longer CNVs than humans. Another possible technical explanation for this observation may be the sequencing libraries used in the two data sets: although Brandler et al. (2016) sequenced most samples with a read length of 100 bp and an average inner distance between reads of 113 bp and others with read lengths of 125 bp and inner distances of 243 bp, the read length of the macaque sequences was larger at 150 bp with an average inner distance of 128 bp. Although we would expect that this difference in read length would allow the macaque calls to be more sensitive to smaller events, the variance in insert size may play a role in the resolution of events that can be detected; unfortunately, we have no information about this variance in the human data. It is also possible that the difference in length distributions is due to a still unidentified technical difference between the two studies.

### De Novo CNVs

In a companion study, we have described the rate and pattern of de novo SNVs in rhesus macaques (Wang et al. 2020). Here, we identify de novo CNVs in the same individuals by looking for CNVs that are unique to the offspring in a trio, as well as being in a heterozygous state. We find only eight total de novo CNVs among our 14 macaque trios, consisting of seven deletions and one duplication (supplementary table S2, Supplementary Material online). The sequence of the duplication is identical in all individuals within the trio, giving us confidence that they are true de novo events. This number of mutations makes the expected number of de novo CNVs 0.29 (95% CI 0.12–0.45) per generation per haploid genome. This rate of mutation is similar to that calculated for humans (Brandler et al. 2016), which is consistent with the similar genome size between the two species. In contrast, the mutation rate of CNVs in *D. melanogaster* was found to be much lower (0.025 per genome; Schrider et al. 2013), though correcting for the ∼30-fold smaller size of the fly genome puts the mutation rates on the same order of magnitude per nucleotide.

**Fig. 3.**
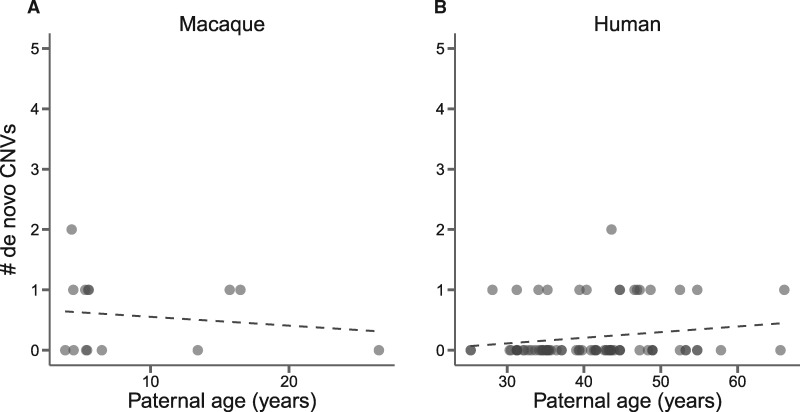
There is no correlation between de novo structural variants in (*A*) 14 macaque trios or (*B*) 97 human trios (12 validated + 5 unvalidated CNVs). Each point represents a single trio.

By considering the age of sires when the offspring of each trio was conceived, we can ask whether the number of de novo CNVs increases in older fathers. We find no paternal age effect in macaques (fig. 3*A*;*R*^2^ = 0.023, df = 12, *P *=* *0.61). Although the age spread of the fathers is skewed young, we find that this does not affect our ability to detect correlations between CNV rates and age (see Materials and Methods), though with only nine events our study has low statistical power to detect an increase. However, we also performed the same analysis using 19 de novo CNVs from human trios (Brandler et al. 2016), and found no increase in the number of mutations in the offspring of older fathers (fig. 3*B*;*R*^2^ = 0.032, df = 77, *P *=* *0.12). Another study of humans with a larger sample size also shows no paternal age effect, with deletions outnumbering duplications among de novo structural variants, confirming the patterns we observe here in both species (Belyeu et al. 2020). Because the rate of new CNVs seems to be very low, increasing the sample size in macaques will increase confidence in our conclusion of a lack of paternal age effect in this species.

### Genomic Context of Macaque CNVs

We find that among the 3,646 CNVs in the macaque samples, 1,609 overlap at least one part of a genic region (table 2). Focusing more closely on likely functional regions of genes, we find that, of those 1,609 CNVs, only 333 overlap at least one exon, meaning that the vast majority of CNVs fall in intergenic or intronic regions. Of the CNVs that overlap an exon, most span more than one, with an average of 5.16 exons per CNV (table 3). However, this is driven by a few CNVs larger than 25 kb that overlap two to three genes. Only 242 CNVs shorter than 25 kb overlap at least one exon, with these 242 overlapping 2.33 exons on an average. In humans, CNVs shorter than 25 kb that overlap at least one exon overlap an average of 5.03 exons.

Among the 1,361 times an exon overlaps with a CNV in macaques, 936 (69%) have been wholly or partially deleted, whereas 425 (31%) have been wholly or partially duplicated (table 1). The ratio of deleted to duplicated exons in macaques is 2.20, which is much lower than the overall ratio of deleted to duplicated regions (7.44). The underrepresentation of deleted exonic regions compared with other regions has been observed previously in primates (Fortna et al. 2004; Dumas et al. 2007; Sudmant et al. 2013) and suggests that gene deletion is more costly in the short-term than duplication. The ratio of deleted-to-duplicated exons in macaques is also significantly lower than the ratio in humans of 2.94 (χ^2^ = 20.82, df = 1, *P* ≪ 0.01). These patterns are consistent among genes, transcripts, and exons (tables 1–3).

**Table 1.** Number of Genic Regions Overlapped by At Least One CNV.

		Genes		10 kb Upstream of Gene		10 kb Downstream of Gene		Transcripts		Exons	
		Del	Dup	Del	Dup	Del	Dup	Del	Dup	Del	Dup
Macaque	Full overlap	100	49	81	38	78	39	145	81	789	392
	Partial overlap	1,112	177	388	109	459	110	2,520	395	147	33
Human	Full overlap	289	166	136	122	109	132	734	422	6,012	2,352
	Partial overlap	5,194	966	2,996	525	3,065	561	21,512	3,754	1,585	235

**Table 2.** Number of CNVs That Overlap At Least One Genic Region.

		Genes		10 kb Upstream of Gene		10 kb Downstream of Gene		Transcripts		Exons	
		Del	Dup	Del	Dup	Del	Dup	Del	Dup	Del	Dup
Macaque	Full overlap	70	37	55	25	54	34	77	38	170	66
	Partial overlap	1,357	200	355	87	404	78	1,357	200	121	24
Human	Full overlap	232	97	88	65	83	73	321	122	1,285	287
	Partial overlap	7,754	1,010	2,766	454	2,860	453	7,754	1,010	982	156

**Table 3.** The Average Number of Genic Regions Overlapped Per CNV for All CNVs and Conditional That the CNV Overlaps at Least One Region.

			Genes		10 kb Upstream of Gene		10 kb Downstream of Gene		Transcripts		Exons	
			Del	Dup	Del	Dup	Del	Dup	Del	Dup	Del	Dup
Macaque	All CNVs	Full overlap	0.038	0.018	0.032	0.012	0.032	0.014	0.053	0.028	0.414	0.211
		Partial overlap	0.388	0.058	0.115	0.036	0.135	0.036	0.872	0.127	0.057	0.011
		All overlaps	0.426	0.076	0.147	0.048	0.167	0.050	0.925	0.155	0.471	0.222
	Conditional on overlapping at least one region	Full overlap	2.000	1.757	2.109	1.760	2.148	1.500	2.506	2.658	8.882	11.65
		Partial overlap	1.041	1.055	1.183	1.506	1.220	1.667	2.343	2.315	1.719	1.708
		All overlaps	1.117	1.260	1.407	1.750	1.410	1.828	2.426	2.575	6.845	9.878
Human	All CNVs	Full overlap	0.019	0.012	0.009	0.009	0.007	0.0098	0.048	0.030	0.689	0.256
		Partial overlap	0.561	0.073	0.225	0.038	0.232	0.039	2.181	0.280	0.161	0.024
		All overlaps	0.580	0.085	0.234	0.047	0.232	0.039	2.228	0.310	0.850	0.280
	Conditional on overlapping at least one region	Full overlap	1.272	1.866	1.591	2.031	1.398	2.000	2.318	3.869	8.325	13.84
		Partial overlap	1.124	1.128	1.266	1.302	1.259	1.347	4.367	4.306	2.547	2.378
		All overlaps	1.147	1.255	1.293	1.503	1.276	1.537	4.408	4.583	6.769	11.37

We find protein-coding transcripts are overlapped 3,141 times by CNVs in macaques, again with an excess of deletions to duplications with a ratio of 5.60. We tested for functional enrichment of these transcripts by examining GO terms. We find 25 GO terms enriched among deleted transcripts in macaques and 28 among duplicated transcripts (supplementary tables S4 and S5, Supplementary Material online). Among these enriched terms are ones related to immune response, ion transport, and nervous system activity. We also note that, of the 1,320 genes that are overlapped by at least one CNV, the vast majority (78%) are only overlapped once. A total of 16 genes are overlapped by more than five unique CNVs (table 4). We identified possible regulatory regions impacted by CNVs by checking for overlaps within 10 kb up- or downstream of a gene. We again find similar patterns of deletions outnumbering duplications in both macaques and humans (table 1).

**Table 4.** Genes Deleted or Duplicated More Than Five Times.

Gene Name	Ensembl ID	Number Deletions	Number Duplications	Gene Function
*SMYD3*	ENSMMUG00000005777	5	2	Histone methyltransferase
*EYS*	ENSMMUG00000041338	6	0	Maintain integrity of photoreceptor cells
*PRKN*	ENSMMUG00000020410	7	1	Protein ubiquitination
*SMOC2*	ENSMMUG00000014474	7	1	Cell adhesiveness
—	ENSMMUG00000037612	2	4	lincRNA
*MUC5B*	ENSMMUG00000010544	6	2	Gel-forming mucin
*LRRTM4*	ENSMMUG00000000181	6	0	Nervous system development
*RASA3*	ENSMMUG00000007434	4	2	Inhibitory regulator of the Ras-cyclic AMP pathway
*IGLV7-43*	ENSMMUG00000031072	5	2	Antigen recognition
Novel gene orthologous to *IGLV1-44*	ENSMMUG00000039568	5	1	Antigen recognition
Novel gene likely in the *IGLV7* family	ENSMMUG00000043547	5	1	Antigen recognition
Novel gene orthologous to *IGLV5-48*	ENSMMUG00000041627	5	4	Probably nonfunctional immunoglobulin
Novel gene orthologous to *IGLV1-47*	ENSMMUG00000040017	5	4	Antigen recognition
*ABR*	ENSMMUG00000008130	6	1	Regulation of GTP-binding proteins
*B3GNTL1*	ENSMMUG00000001110	5	2	Putative glycotransferase
Novel gene orthologous to *SHC2*	ENSMMUG00000000485	5	1	Signaling adaptor in cortical neurons

Note.—Orthologs reported are from humans. Gene functions obtained from UniProt.

### Gene Duplications and Losses within and between Species

**Fig. 4.**
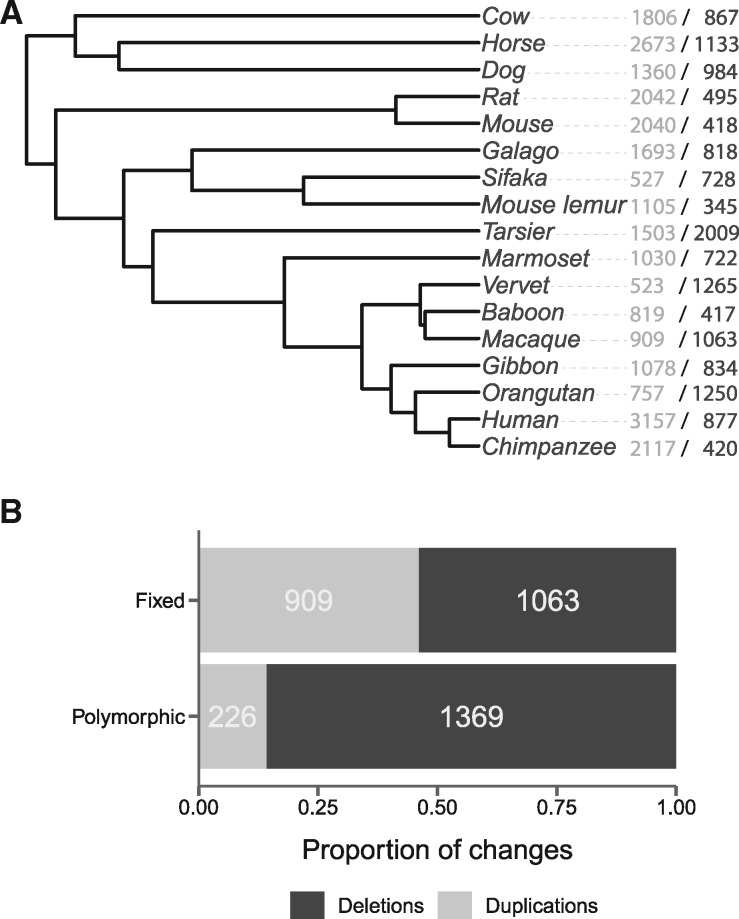
(*A*) Long-term patterns of gene gain and loss were inferred for macaques by comparing gene copy numbers among 17 mammal species. The number of genes gained or lost is shown for each tip lineage in the format: # gained/# lost. (*B*) Among genes in both the gene family (Fixed) and CNV (Polymorphic) analyses, we find that genes are more likely to be part of polymorphic deletions, and conversely that there is a larger proportion of duplications among fixed differences.

The ultimate fate of structural variants is to either become fixed in a population or to be lost. Genes overlapping CNVs can play a role in this process by conveying fitness benefits or costs depending on their copy number. We investigated the long-term fate of genes involved in copy-number variation in macaques using gene gains and losses among 17 mammal species (fig. 4*A*). By comparing the number of genes gained and lost between species to the number of genes overlapping segregating CNVs within macaques, we hope to reveal patterns in the long-term evolution of gene copy number.

We analyzed copy-number variation of genes in 10,798 gene families across 17 species (fig. 4*A* andsupplementary table S6, Supplementary Material online). We estimate that for 13 of the 17 tip species gene duplications exceed gene deletions. Along the branch leading to macaques since their common ancestor with baboons (∼11 Ma), we infer the loss of 1,063 and the gain of 909 protein-coding genes, respectively, for a loss-to-gain ratio of 1.17 (fig. 4*B*). Both of these patterns are in contrast to the short-term estimates of copy-number polymorphism for both humans and macaques, which both show a large excess of gene deletions. The skew toward polymorphic deletions suggests either that purifying selection is acting on deletions—and therefore our polymorphism data are detecting events that will not be fixed—or that positive selection is acting on duplications, causing them to fix at higher rates. If selection is playing a role in the decreased frequency of deletions over time, we may not expect to observe the extreme bias toward deletions in polymorphic data that we see, since it is likely deletions under purifying selection would be eliminated quickly. However, selection on CNVs may be more evident in coding regions. To test this, we compared the total number of CNVs of each type (deletion or duplication) to the number that overlap exons. We find that the ratio of deletions to duplications within exons in macaques (3.2; table 2) is significantly less than what we would expect from the ratio across the genome as a whole (7.4; χ^2^ = 41.344, df = 1, *P* ≪ 0.01). The same is true of the human data (χ^2^ = 50.362, df = 1, *P* ≪ 0.01). This indicates that, although deletions may be the more common event throughout the genome, they are less likely to arise and persist in coding regions. It should be noted that these results come from observations of two species in this study, so it remains to be seen if this pattern is general across primates.

The loss-to-gain ratio along the macaque branch is only a third of that observed among protein-coding transcripts overlapped by segregating CNVs (see above), but could be biased because different genes may be included in the different annotation sets used. Restricting our CNV analysis to the 21,059 protein-coding transcripts used in the gene family analysis, we find a ratio of 6.06 deletions to duplications, still significantly higher than the long-term ratio of gene gain to loss (fig. 4*B*; χ^2^ = 412.82, df = 1, *P *≪* *0.01). Together, these results indicate that, although deletions dominate among de novo mutations and segregating CNVs in macaques, the number of genes gained and lost is more balanced over evolutionary timescales.

## Discussion

Copy-number variation can play a key role in disease and evolution (Eichler et al. 2007; Zhang et al. 2009; Girirajan et al. 2011). Here, we have shown that patterns of copy-number variation in rhesus macaques are largely similar to humans: segregating CNVs in both species are overwhelmingly made up of deletions. CNVs in macaques appear to be on an average longer than in humans, though this may also be the result of an unidentified methodological bias. We found that de novo CNVs show no correlation with parental age in either species. This is in contrast to SNVs, which have been found to increase with paternal age in both species (Kong et al. 2012; Jonsson et al. 2017; Wang et al. 2020). The difference between SNVs and CNVs is likely due to the differences in how these mutations arise. SNVs are thought to arise as errors in the DNA replication process during mitosis, or more rarely as unrepaired damage to DNA caused by the environment (Crow 2000). For male mammals, both of these processes are ongoing throughout the lifetime, with recurring mitoses occurring during spermatogenesis. However, copy-number variation is thought to arise only during unequal cross-over events during meiosis (Hastings et al. 2009; Zhang et al. 2009). Since meiosis occurs only once per generation, we expect no age effects for mutations that arise from it. This expectation is consistent with our present observations in macaques and previous studies in humans (MacArthur et al. 2014; Kloosterman et al. 2015).

With no age effect for copy-number mutations, we expect the rate of new CNVs per unit time (i.e., year) to be subject to a classic generation-time effect (Laird et al. 1969; Wu and Li 1985). The generation-time effect posits that species with shorter generation times accumulate more mutations over time because they experience more germline cell divisions per unit time. This generation-time effect has been found to be dampened for single-nucleotide mutations, which are dependent on mitosis, because of ongoing spermatogenesis (Thomas and Hahn 2014). However, for structural variants that occur during meiosis this effect should hold for neutral changes. The generation-time effect is a life-history model that provides a useful expectation when comparing rates of copy-number variation between species. Under this model, we would expect rhesus macaques, with shorter generation times, to have a higher rate of long-term copy number evolution than humans if life-history traits are the only factor determining rates of copy-number changes.

Contrary to these expectations, the reverse relationship has been observed between species, with humans and chimps having the highest rate of gene gain and loss among primates (Hahn et al. 2007; Marques-Bonet et al. 2009). One possible explanation for the discrepancy between the expected and observed rate patterns of genic copy-number variation between these two species is a difference in selection between them. In this scenario, the underlying mutation rates per unit time differ, but studies of genic copy-number variation reveal the combined effects of mutation and selection in shaping the accumulation of change. In support of this is our observation in macaques that deletions make up the majority of polymorphic copy number events throughout the genome, but polymorphisms that overlap exons and fixed gene gains and losses are more evenly balanced when comparing gene copy-number evolution between species. This is also further evidence for the claim that deletions are under stronger purifying selection than duplications (Conrad et al. 2006; Schrider and Hahn 2010; Schrider et al. 2013).

Taken together, the patterns of copy-number variation we have uncovered will help to develop models of this type of mutation and to determine the prevailing drivers of long-term structural variant evolution. Comparisons of variants between humans and model organisms such as macaques can inform us about the suitability of these models for the study of certain types of disease. Our comparisons show that macaques and humans have similar short-term patterns of structural variation, but that these patterns diverge on longer timescales. Although the patterns uncovered here provide a strong basis for these conclusions, larger samples in future studies will provide better estimates of important parameters. In addition to helping refine disease models, the rates of de novo CNV mutation are an important clue to determining the processes governing the evolution of the mutation rate. For the types of structural variation studied here, we find no difference in rates of de novo mutation between humans and macaques, indicating a common mechanism for CNV generation that is likely driven by the single meiosis event that occurs in the germline of both species. Ultimately, understanding the selective forces on different developmental and evolutionary timescales will require tracking variants at each stage from introduction to fixation.

## Materials and Methods

### Sequencing and Read Mapping

About 32 rhesus macaque individuals were chosen from available pedigrees at the California National Primate Research Center (supplementary table S1, Supplementary Material online). Genomic DNA was isolated from blood samples of these animals for whole-genome sequencing (Illumina Nova-Seq, average 40× average coverage). Reads were mapped to the reference macaque genome (rheMac8.0.1, GenBank assembly accession number GCA_000772875.3) using BWA-MEM version 0.7.12-r1039 (Li 2013) to generate a BAM file for each individual. Duplicate reads were identified with Picard MarkDuplicates version 1.105 (http://broadinstitute.github.io/picard/; last accessed December 2, 2020) and these reads were excluded from subsequent analyses. All BAM files were sorted and indexed with samtools version 1.9 (Li et al. 2009).

Reads that map to the reference with unexpected distances given their insert size (split reads) or orientations (discordant reads) between mate pairs can be used as signals of genomic deletion and duplication. These split and discordant reads were identified in each individual with samtools version 1.9 (-F 1294 for discordant reads) and the extractSplitReads_BwaMem script included in the Lumpy (Layer et al. 2014) software package. This resulted in three BAM files for each individual used as input for the CNV calling software listed below: all reads, discordant reads, and split reads.

### Calling CNVs in Rhesus Macaques

Copy-number variants were called only on contigs that map to assembled macaque chromosomes. We used a suite of methods in the SpeedSeq software (Chiang et al. 2015) that use patterns of split and discordant read mappings to identify structural variant breakpoints throughout the genome to call CNVs. First, Lumpy (Layer et al. 2014) was used to find putative breakpoint sites in all 32 macaque individuals. Lumpy uses several pieces of evidence (such as split and discordant reads) to probabilistically model where breakpoints occur in the genome. CNVs called by Lumpy were genotyped with SVtyper (Chiang et al. 2015), which uses a Bayesian framework much like that used to genotype SNVs to determine whether CNVs are homozygous or heterozygous. For CNV calling with Lumpy, repetitive regions were masked using the rheMac8 RepeatMasker table from the UCSC table browser (Karolchik et al. 2004; http://genome.ucsc.edu/).

The software SVtools (Larson et al. 2018) was used to combine the calls from the 32 individuals into a single set. This set was then regenotyped with SVtyper to obtain information for all CNVs in all samples (even if they were not present in that sample) for filtering. CNV calls were annotated with read depth information using Duphold (Pedersen and Quinlan 2019) and finally, CNVs were pruned with SVtools such that, among events found to occur within 100 bp, only the event with the highest quality score was retained. CNVs were then annotated as to their overlap with genes by using the UCSC table browser. GNU Parallel (Tange 2011) was used throughout to parallelize the CNV calling software across individuals.

### Filtering Putative Macaque CNVs

The process for calling CNVs resulted in 157,914 events at 8,515 sites. To reduce the number of false positives, we applied the following filters to our set of CNVs:

1. Removed 83,371 CNVs at 2,615 sites that are present in at least 31 of the 32 individuals. These are most likely events in the reference individual, or misassemblies.
2. Removed 4,934 CNVs at 464 sites over 100,000 bp in length.
3. Removed 435 CNVs at 244 sites with a quality score <100.
4. Retained only deletions in which the fold-change of read depth for the variant is <0.7 of the flanking regions. This filter removed 12,763 CNVs at 870 sites.
5. Retained only duplications in which the fold-change of read depth for the variant is >1.3 of regions with similar GC content. This filter removed 9,954 CNVs at 568 sites.
6. Removed 2,167 CNVs at 108 sites between 275 and 325 bp to filter out putative Alu elements.

These filters yield a reduced CNV call-set of 44,290 events at 3,646 sites which was used for all subsequent analyses (supplementary table S2, Supplementary Material online).

### Identifying De Novo CNVs and Calculating the Mutation Rate

From the full set of 3,6464 CNVs, we identified de novo events as those that occur only in one of the probands of the 14 trios. We required both parents to be homozygous for the reference allele and the child to be heterozygous. For F_1_ probands, the de novo CNV was allowed to be present in the proband’s offspring, as new mutations would be expected to be transmitted roughly half the time. This occurred in two out of the three F_1_ CNVs.

We calculated the CNV mutation rate per generation for a haploid genome by taking the mean number of transmissions in the 14 macaque trios and dividing by 2. Standard error for this rate was calculated by taking the standard deviation of the number of transmissions for the 14 trios divided by the square root of the number of trios times a critical value of 1.96 for the 95% confidence interval.

We conducted a power analysis to show that the age variation seen in our sample does not prevent us from detecting the positive accumulation of mutations across the macaque lifespan. We used our data on SNV mutations (Wang et al. 2020) as an example of the ability to detect a positive association with parental age, given the distribution of ages in our sample. Using the age coefficient from a linear model of mutations with age, we simulated new mutation counts for each trio under a Gaussian model of error variance. We found a significant positive age coefficient in 9,992 out of 10,000 simulations at the *P* < 0.01 level (i.e., our statistical power is >99%). We also simulated mutation counts under a Poisson model. In this case, each of the age coefficients in all 10,000 simulations were significant and positive at the *P* < 0.01 level. The amount of age variation in our sample is therefore sufficient to significantly detect an age effect on CNV mutations if they accumulate at the same rate estimated from the SNV data.

### Human CNV Data

Human CNVs were downloaded from the supplemental material of Brandler et al. (2016). This study used 235 individuals in 69 families to look for patterns of de novo structural variation among autism patients. Their de novo mutations along with parental ages were obtained from their supplementary spreadsheet S1, Supplementary Material online and used for figure 3*B*. The entire CNV call-set from their supplementary data S1, Supplementary Material online was used for all other comparisons. These authors used two methods to call CNVs, Lumpy (Layer et al. 2014) and ForestSV (Michaelson and Sebat 2012), and two methods to genotype their CNV calls, SVtyper (Chiang et al. 2015) and gtCNV (now known as SV^2^; Antaki et al. 2018). We restrict our comparisons to those called with Lumpy and genotyped with SVtyper for consistency with our methods. We also exclude calls from this set between 275 and 325 bp in length as putative Alu elements. For de novo mutations, we include both validated and unvalidated CNVs, however our results remain the same when excluding the unvalidated calls (supplementary fig. S7, Supplementary Material online).

### Counting Overlaps between CNVs and Genomic Regions

We annotated CNVs based on overlaps with genic regions for both the human and macaque data. Genome annotations were downloaded for both species in the form of GTF files from Ensembl (release 97 for the macaque data and release 84 for the human data, coinciding with publication of the human CNV calls). Coordinates for each CNV were then cross-checked with coordinates for genes, transcripts, and exons to determine how many times a CNV overlaps a genic region (tables 1–3) using bedtools (Quinlan and Hall 2010). In the absence of annotated regulatory regions for macaques, regions 10 kb up- and downstream of genes were taken as proxies for regulatory regions and counted as well (tables 1–3). GO terms for transcripts were downloaded from Ensembl. A Fisher’s exact test was performed on terms annotated to transcripts that overlap a CNV vs. those that do not, with a false discovery rate of 0.01 (supplementary tables S4 and S5, Supplementary Material online).

### Counting Fixed Macaque Gene Duplications and Losses

In order to identify genes gained and lost on the macaque lineage, we obtained peptides from human, chimpanzee, orangutan, gibbon, macaque, vervet, baboon, marmoset, tarsier, mouse lemur, sifaka, galago, rat, mouse, dog, horse, and cow from ENSEMBL 95 (Zerbino et al. 2018). To ensure that each gene was counted only once, we used only the longest isoform of each protein in each species. We then performed an all-vs-all BLAST (Altschul et al. 1990) on these filtered sequences. The resulting e-values were used as the main clustering criterion for the MCL program to group peptides into gene families (Enright et al. 2002). This resulted in 15,662 clusters. We then removed all clusters only present in a single species, resulting in 10,798 gene families. We also obtained an ultrametric tree (fig. 4*A*) from a previous study (Rogers et al. 2019) for 12 mammal species and added mouse lemur (Larsen et al. 2017), tarsier, vervet, and galago based on their divergence times from timetree.org (Kumar et al. 2017).

With the gene family data and ultrametric phylogeny as input, we estimated gene gain and loss rates with CAFE v4.2 (Han et al. 2013) using a three-rate model, which has been shown to best fit mammalian data (Hahn et al. 2007; Marques-Bonet et al. 2009; Carbone et al. 2014). CAFE uses the estimated rates to infer ancestral gene counts and we subsequently counted the number of genes gained and lost in the macaque lineage relative to its common ancestor with baboon.

## Supplementary Material

Supplementary data are available at *Molecular Biology and Evolution* online.

## Supplementary Material

msaa303_Supplementary_Data
