# Supplementary material for: Origins and Long-Term Patterns of Copy-Number Variation in Rhesus Macaques
Source: Mol Biol Evol. 2020 Nov 23;38(4):1460–71. doi: 10.1093/molbev/msaa303 (PMC8042740; doi:10.1093/molbev/msaa303)
Supplement: msaa303_Supplementary_Data [file msaa303_supplementary_data.zip › FiguresS1-7.pdf]

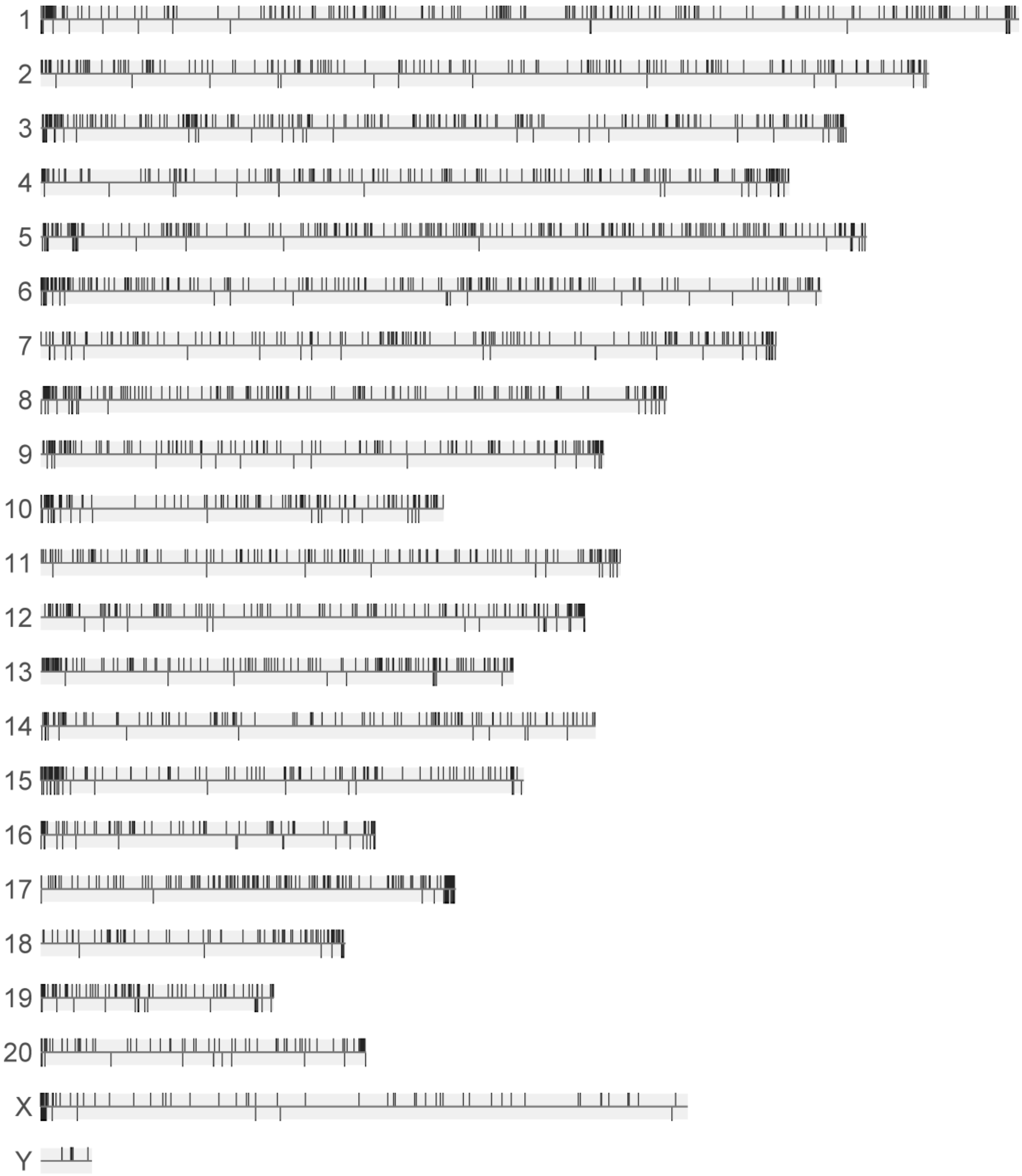

Figure S1: Locations of identified CNVs on the 22 rhesus macaque chromosomes. Deletions extend above the horizontal line for each chromosome and duplications extend below it.

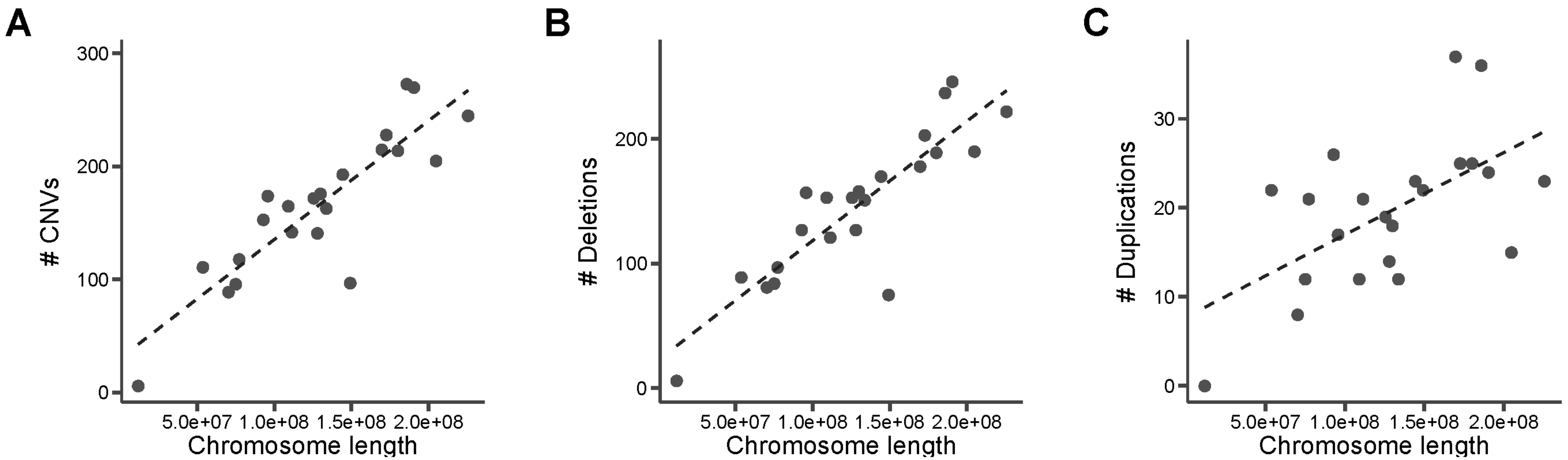

Figure S2: The number of CNVs is strongly correlated with chromosome length in macaques for **(A)** all CNVs, **(B)** deletions, **(C)** and duplications.

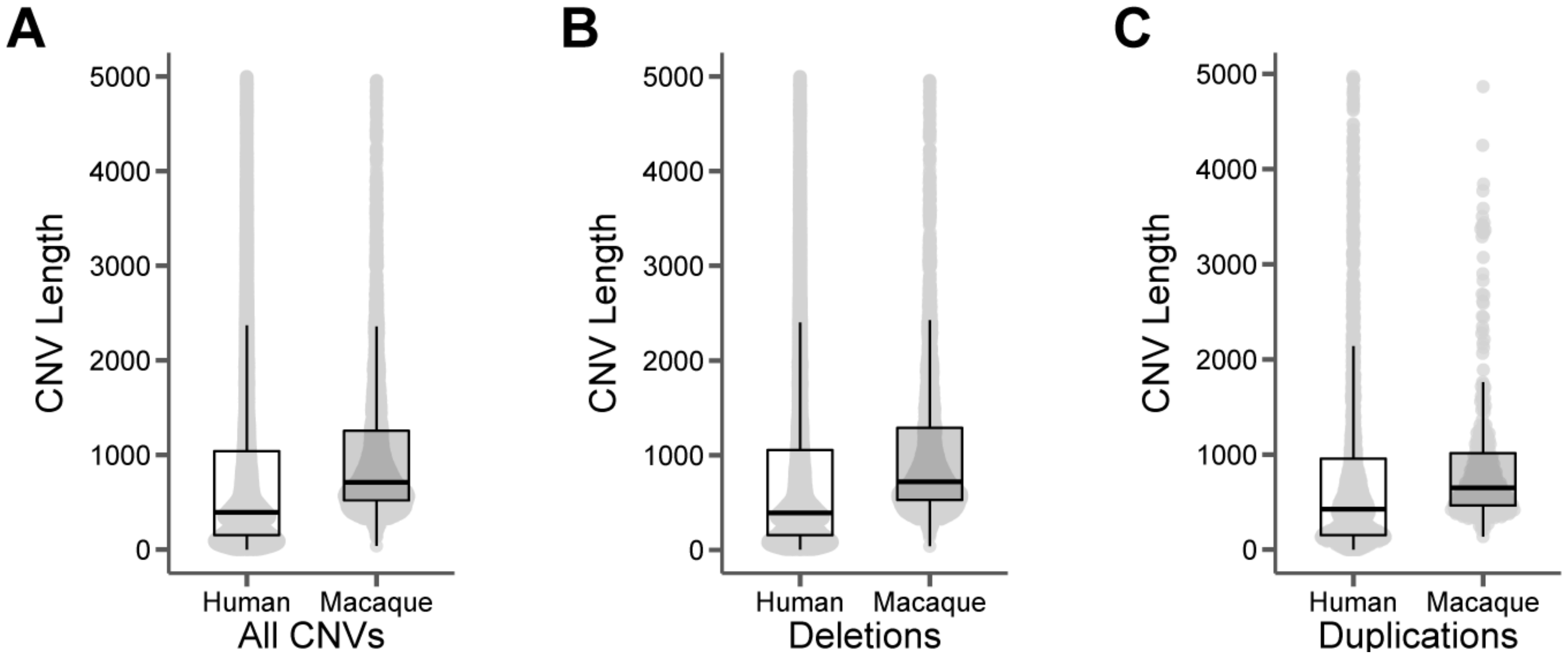

Figure S3: Length distributions of CNVs shorter than 5000 bases and excluding all calls between 275 and 325bp long as possible unannotated Alu elements. Distributions shown for (**A**) all CNVs, (**B**) deletions only, and (**C**) duplications only. Macaque CNVs are longer on average for each class.

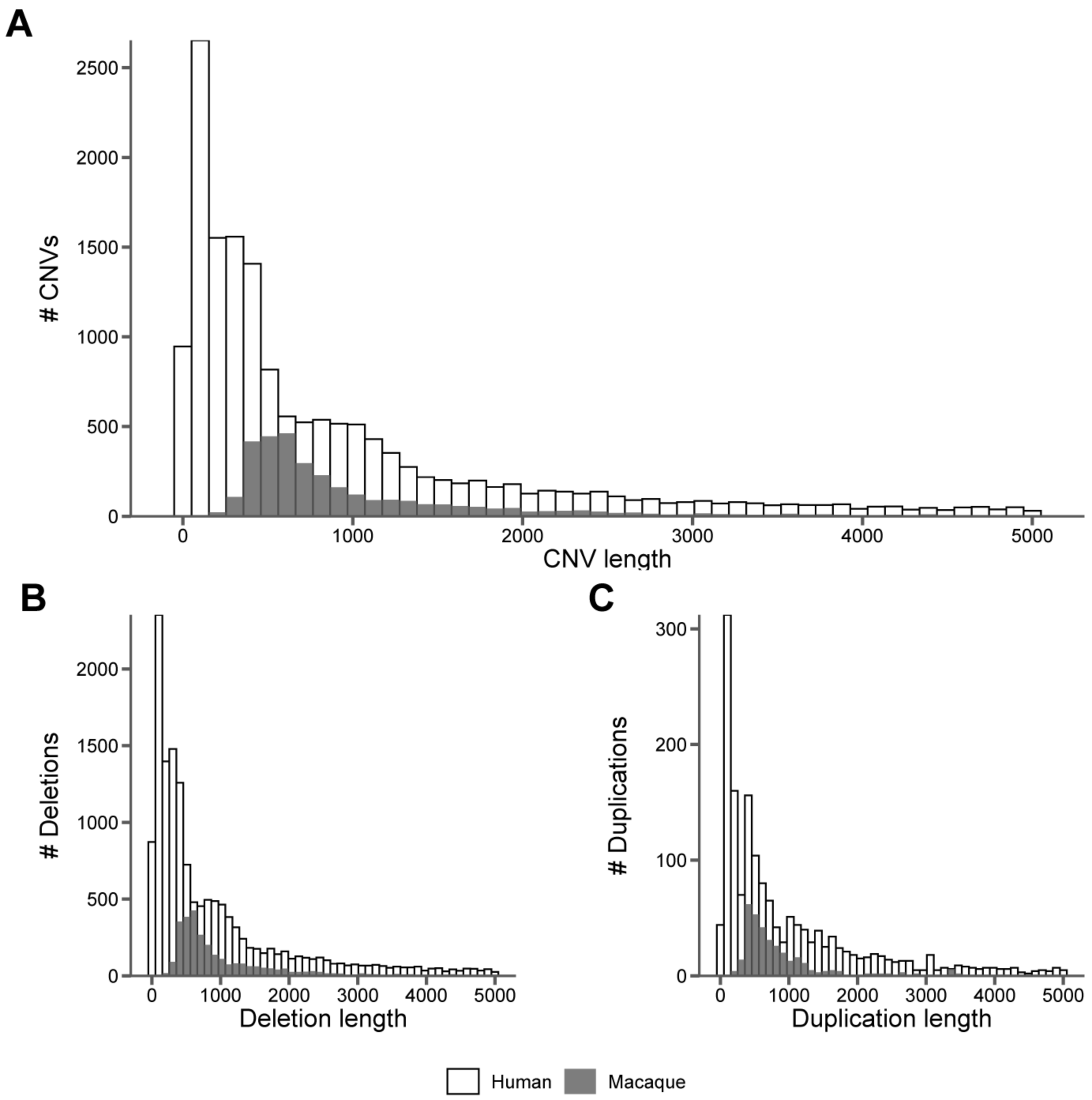

Figure S4: Length distributions of CNVs shorter than 5000 bases using the full human CNV dataset. Macaque CNVs are longer on average than humans for (**A**) all CNVs (Kolmogorov-Smirnov $D = 0.38$, $p \ll 0.01$), (**B**) deletions only (Kolmogorov-Smirnov $D = 0.39$, $p \ll 0.01$), and (**C**) duplications only (Kolmogorov-Smirnov $D = 0.31$, $p \ll 0.01$). Values are overlaid, with macaque bins in front of human bins. All bins start at 0 on the y-axis.

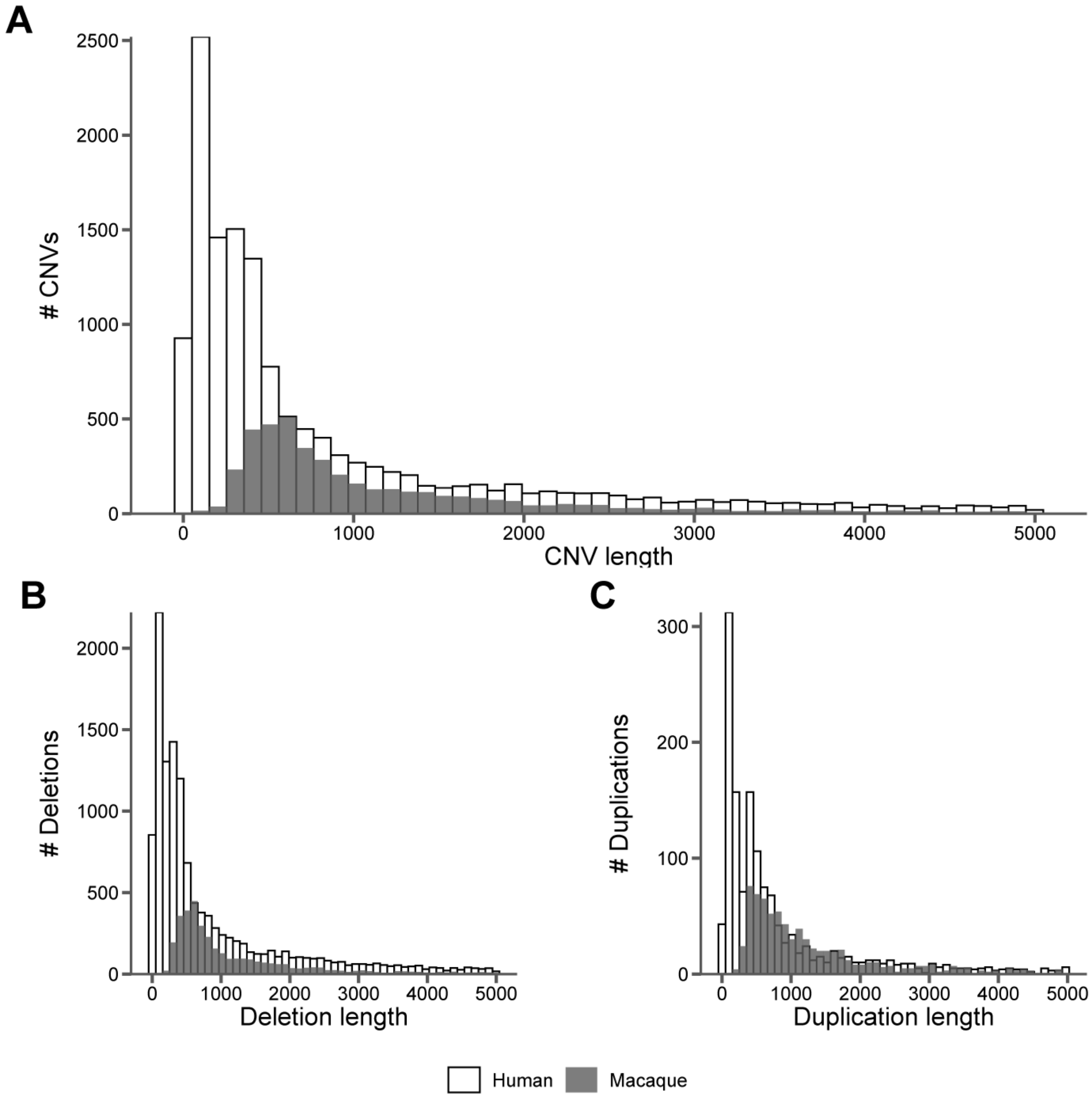

Figure S5: Length distributions of CNVs shorter than 5000 bases using the unfiltered macaque call set. Macaque CNVs are longer on average than humans for (**A**) all CNVs (Kolmogorov-Smirnov $D = 0.40$, $p << 0.01$), (**B**) deletions only (Kolmogorov-Smirnov $D = 0.41$, $p << 0.01$), and (**C**) duplications only (Kolmogorov-Smirnov $D = 0.39$, $p << 0.01$). Values are overlaid, with macaque bins in front of human bins. All bins start at 0 on the y-axis.

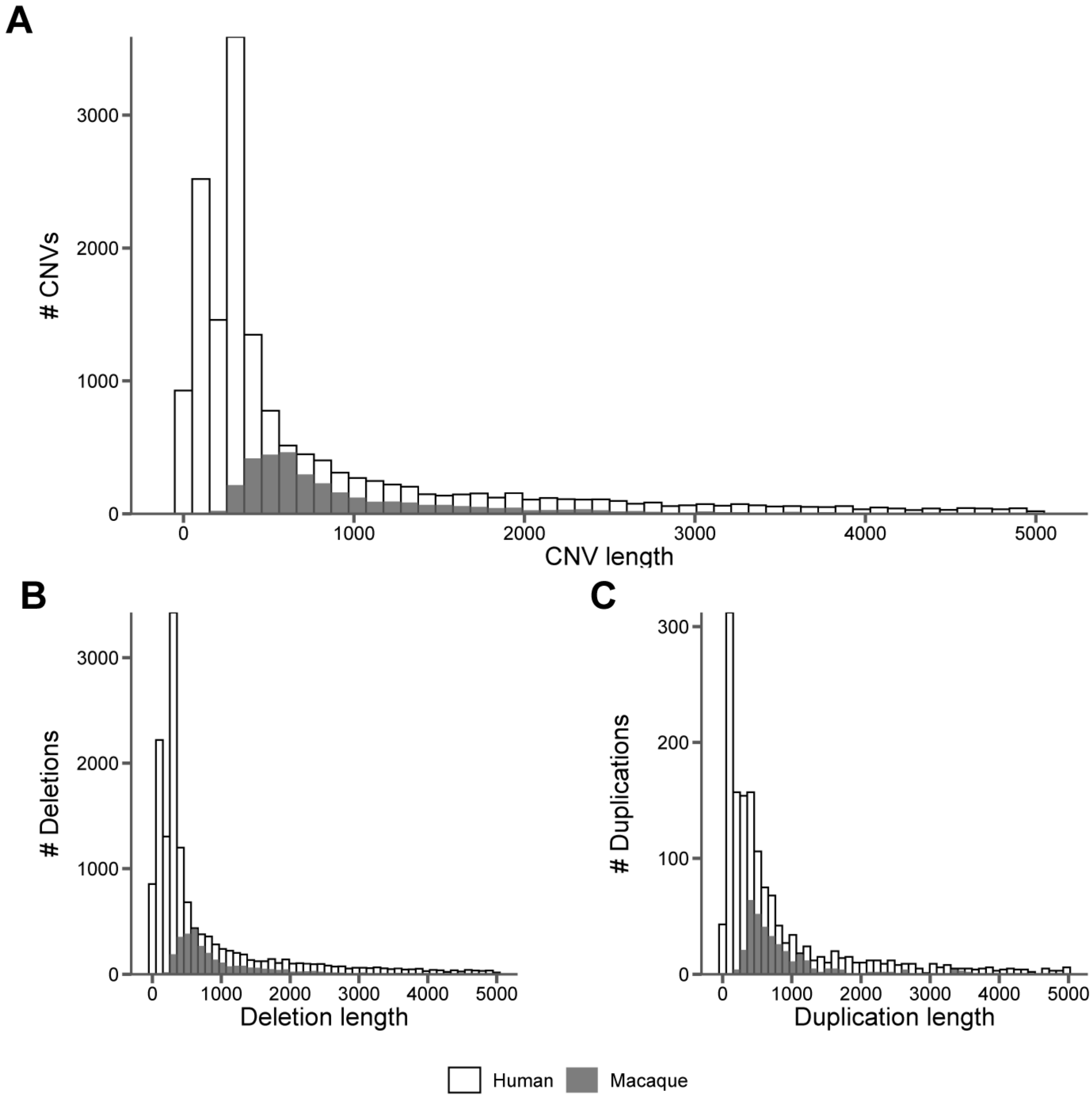

Figure S6: Length distributions of CNVs shorter than 5000 bases without excluding all calls between 275 and 325bp long in order to exclude possibly unannotated Alus. Macaque CNVs are longer on average than humans for (**A**) all CNVs (Kolmogorov-Smirnov $D = 0.46$, $p << 0.01$), (**B**) deletions only (Kolmogorov-Smirnov $D = 0.47$, $p << 0.01$), and (**C**) duplications only (Kolmogorov-Smirnov $D = 0.38$, $p << 0.01$). Values are overlaid, with macaque bins in front of human bins. All bins start at 0 on the y-axis.

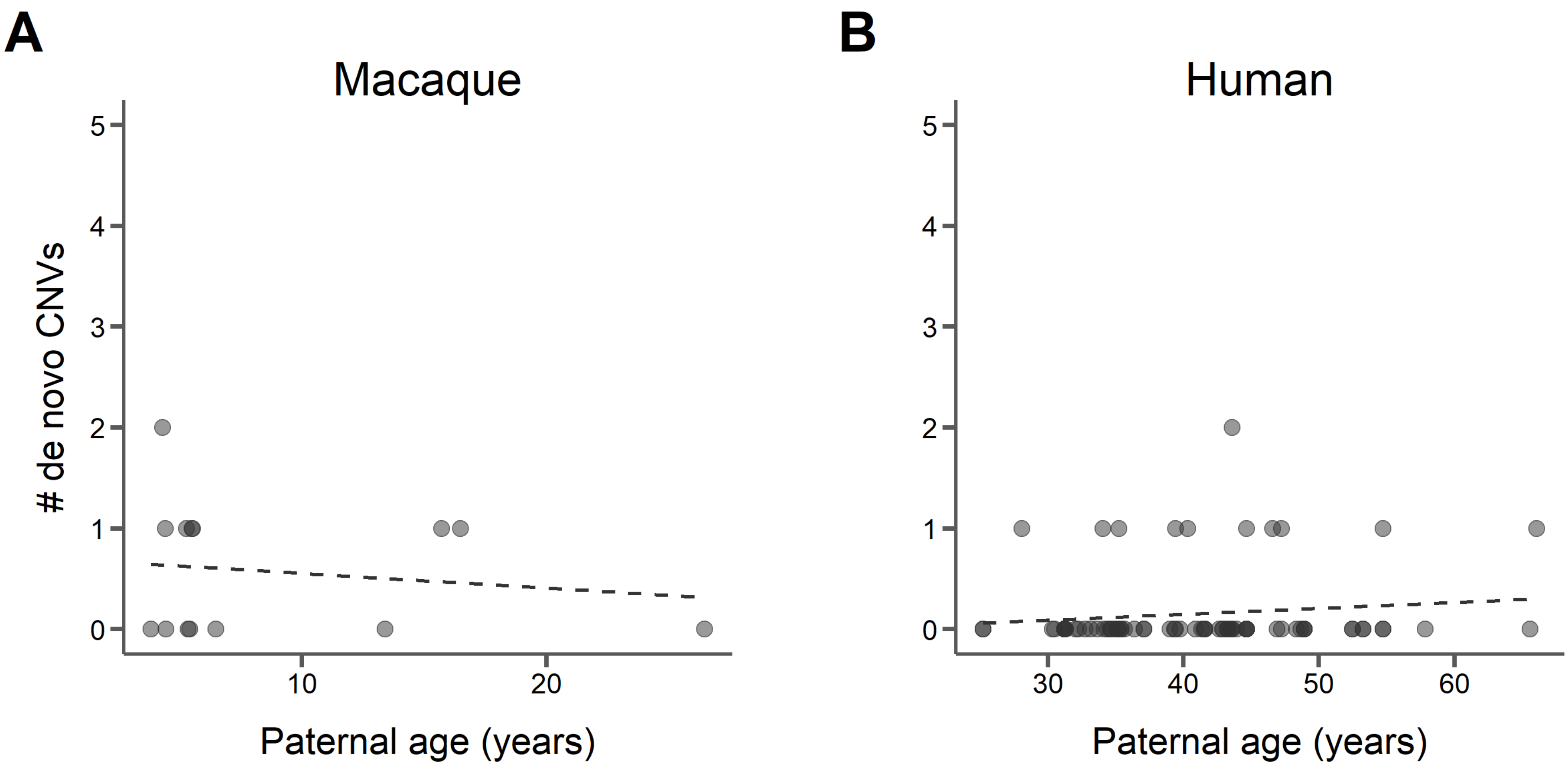

Figure S7: There is no correlation between *de novo* structural variants in (**A**) 14 macaque trios or (**B**) 97 human trios (12 validated CNVs only). Each point represents a single trio.
